# Milligan-Morgan hemorrhoidectomy combined with non-doppler hemorrhoidal artery ligation for the treatment of grade III/IV hemorrhoids: a single centre retrospective study

**DOI:** 10.1186/s12876-023-02933-x

**Published:** 2023-08-31

**Authors:** Qing Long, Yong Wen, Jun Li

**Affiliations:** Department of Traditional Chinese Medicine, The Affiliated Hospital of Southwest Medical University, Luzhou, 646000 Sichuan China

**Keywords:** Milligan-Morgan hemorrhoidectomy (MMH), Non-Doppler hemorrhoidal artery ligation (ND-HAL), Hemorrhoids, Complication

## Abstract

**Background:**

Milligan-Morgan hemorrhoidectomy (MMH) is the most widely used surgical procedure because of its precise curative effect, but it has the disadvantages such as obvious postoperative pain and bleeding. To retrospectively evaluate the efficacy and safety of MMH combined with non-Doppler hemorrhoidal artery ligation (MMH + ND-HAL) for the treatment of grade III/IV hemorrhoids.

**Methods:**

We conducted a retrospective analysis of 115 patients with grade III/IV hemorrhoids, 53 patients had received MMH + ND-HAL, and the remaining 62 patients received MMH. We collected and compared demographic and clinical characteristics of both groups, including intraoperative blood loss, postoperative visual analog scale (VAS) for pain, analgesic consumption, postoperative bleeding, perianal incision edema, urinary retention, anal stenosis, anal incontinence incidence, recurrence rate (prolapse or bleeding), and patient satisfaction.

**Results:**

The VAS pain score of the first postoperative defecation and at the postoperative 12 h, 1 day, 2 days, 3 days, and 7 days, as well as the total analgesic consumption within 7 days, for the MMH + ND-HAL group were lower than those for the MMH group (*P* < 0.05). The intraoperative blood loss, the incidence of postoperative bleeding, perianal incision edema, and urinary retention in the MMH + ND-HAL group was lower than that in the MMH group (*P* < 0.05). No anal stenosis or anal incontinence occurred in either group. At follow-up by telephone or outpatient 12 months after surgery, the recurrence rate (prolapse or bleeding) was lower in the MMH + ND-HAL group than in the MMH group (*P* < 0.05), and satisfaction was higher in the MMH + ND-HAL group than in the MMH group (*P* < 0.05).

**Conclusions:**

MMH + ND-HAL was a satisfactory surgical modality for treating III/IV hemorrhoids.

## Background

Hemorrhoids are one of the most common anorectal diseases [1, 2] and are generally caused by the weakening of the anal cushion and the supporting tissue and spasms of the internal sphincter [3]. Hemorrhoids can occur at different ages, and the prevalence of hemorrhoids in adults is 11% [4]. With an increase in age, the incidence rate gradually increases, which seriously affects people’s quality of life. The main symptoms of hemorrhoids are bleeding, prolapsing, pain, swelling, itching, and mucous soiling. Although hemorrhoids are not malignant, the symptoms of hemorrhoids can have negative psychological and physical effects on patients, and even may induce secondary anemia or massive bleeding that could threaten the life and health of patients [5, 6].

Grade III/IV hemorrhoids according to Goligher’s classification often require surgical treatment [7, 8]. In recent years, various surgical operations have been used to treat symptomatic hemorrhoids. Hemorrhoidectomy is the first choice for patients with grade III/IV hemorrhoids because of its clear effect and high success rate [9, 10]. Milligan-Morgan hemorrhoidectomy (MMH) is one of the most widely used and representative operations. However, the procedure is associated with postoperative pain and bleeding [11], which is the main reason why patients are afraid and reluctant to undertake the procedure [12].

To reduce the occurrence of pain, bleeding, and other complications after MMH, we adopted MMH combined with non-Doppler hemorrhoidal artery ligation (MMH + ND-HAL), a new combined operation, to overcome some limitations of MMH. The key characteristic of this combined operation mode was that it not only could remove hemorrhoid tissue but also reduced the occurrence of severe incision pain and bleeding and other complications after MMH. At present, no data are available to compare the efficacy and safety of MMH + ND-HAL and MMH; thus, the purpose of this article was to retrospectively compare and analyze the effectiveness and safety of these two operations.

## Materials and methods

### Patients

We retrospectively analyzed 115 cases of patients with grade III/IV hemorrhoids who received surgical treatment in the Affiliated Hospital of Southwest Medical University between March 2019 and March 2021, of which 53 patients received MMH + ND-HAL, and the remaining 62 patients received MMH. The inclusion criteria of this study were as follows: (1) grade III/IV hemorrhoids (Goligher’s classification); and (2) age 18 to 65 years old, regardless of gender. The exclusion criteria were as follows: (1) accompanied by other anorectal diseases, such as perianal abscess, fistula, anal fissure, or inflammatory bowel disease; (2) patients with hypertension, diabetes, or abnormal liver and kidney functions; (3) patients who previously underwent hemorrhoid surgery; and (4) patients with coagulation dysfunction. This study was approved by the Ethics Committee of the Affiliated Hospital of Southwest Medical University. All operations were performed by experts with senior professional titles in anorectal surgery.

### Data collection

All relevant data saved in the computer database after operation were collected retrospectively. The following parameters were recorded and analyzed: age, sex, grade of hemorrhoids, duration of disease, intraoperative blood loss, postoperative visual analog scale (VAS) for pain at each time point after surgery (the first defecation after surgery, as well as the 12th hour, 1st day, 3rd day, and 7th day after surgery), and total analgesic consumption within 7 days. We collected data on the incidence of postoperative complications, including minor bleeding, perianal incision edema, acute urinary retention, anal stenosis, and anal incontinence. After the operation, patients were rechecked in the anorectal clinic of our hospital every week until they were fully recovered. Follow-up was conducted by telephone or outpatient at 12 months to assess the recurrence (prolapse or bleeding) rate and patient satisfaction. Patient satisfaction was evaluated on a 5-point scale of very dissatisfied, somewhat dissatisfied, normal, somewhat satisfied, and very satisfied. “Satisfaction” was defined as the sum of somewhat satisfied and very satisfied.

### Surgical procedures

**Fig. 1:**
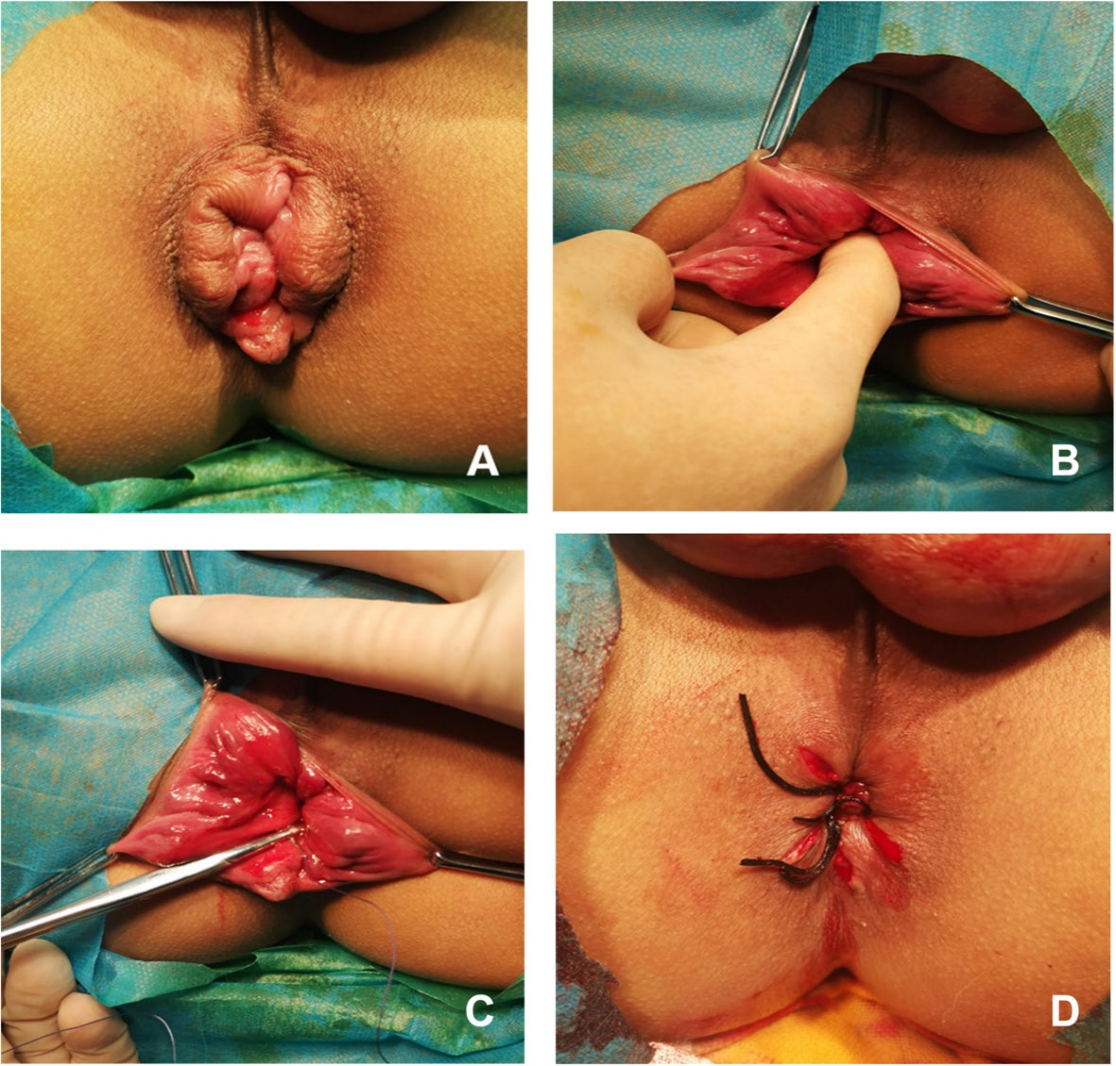
**A** The patient was placed in the lithotomy position, and the distribution of hemorrhoids was observed after anal enlargement. **B** Using the index finger to find the pulsating hemorrhoidal artery. **C** Exposing hemorrhoids with allis forceps, and ligating the pulsating hemorrhoidal artery with 2 − 0 absorbable suture. **D** MMH was performed

The MMH + ND-HAL procedure was performed according to the following steps: (1) The patients received spinal anesthesia and were placed in the lithotomy position; the anal canal and the lower end of the rectum were disinfected with 0.5% iodophor; and the distribution of hemorrhoids after anal dilatation was observed (Fig. 1A). (2) About 2–3 cm above the dentate line, the index finger was used to find the pulsating hemorrhoidal artery (Fig. 1B). (3) The hemorrhoids were exposed with allis forceps, and the pulsating hemorrhoidal arteries were ligated with 2 − 0 absorbable suture. Generally, the ligation position was 3, 7, or 11 o’clock points above the dental line. The ligation depth could not be too shallow or too deep, and the degree was submucosa (Fig. 1C). (4) MMH was performed according to the standard technique described by Milligan and Morgan [13] (Fig. 1D).

### Postoperative management

Postoperative management included the prohibition of food and water, lying flat for 6 h, stool control for 24 h, intravenous drip of antibiotics (cefuroxime) for 2 days to prevent infection, clean anus, and change of dressing after defecation. When the pain of the patient was intolerable, the oral analgesic nimesulide dispersible tablets (0.1 g/tablet) were given and the dose was recorded.

### Statistical analysis

We used SPSS version 25.0 (SPSS Inc., Chicago, IL, USA) for analysis. Continuous variables were expressed as mean ± standard deviation, and the t-test was performed. We analyzed categorical variables using the Pearson chi-square test or Fisher’s exact test. The data were regarded as statistically significant when *P* < 0.05.

## Result

### Patient characteristics

We enrolled a total of 53 patients in the MMH + ND-HAL group (among which 45 patients had grade III hemorrhoids, and 8 patients had grade IV hemorrhoids) and 62 patients in the MMH group (52 patients had grade III hemorrhoids, 10 patients had grade IV hemorrhoids). There were no significant differences in age, sex, duration of disease, hemorrhoid grade, or number of surgical incisions between the two groups, but intraoperative bleeding in the MMH + ND-HAL group was less than in the MMH group (*P* < 0.05) (Table 1).

**Table 1:** Patient characteristics

Group	MMH + ND-HAL (*n* = 53)	MMH (*n* = 62)	t/χ 2 value	*P*-value
Age (years)	44.02 ± 10.67	42.24 ± 9.91	0.950	0.344
Male/female	32/21	37/25	0.006	0.939
duration of disease (years)	6.43 ± 4.76	5.79 ± 4.15	0.847	0.399
Grade of hemorrhoids (III/IV)	45/8	52/10	0.023	0.879
Intraoperative blood loss (mL)	7.43 ± 2.65	8.69 ± 3.33	−2.217	

Age, duration of disease, and number of hemorrhoids excised are presented as the mean ± standard deviation

*MMH + ND-HAL* Milligan-Morgan hemorrhoidectomy combined with non-Doppler hemorrhoidal artery ligation and perianal sealing, *MMH *Milligan-Morgan hemorrhoidectomy

### Complications

In the MMH + ND-HAL group, postoperative visual analog scale (VAS) for pain at the first defecation, 12 h, 1 day, 2 days, 3 days, and 7 days after operation were lower than those in the MMH group (*P* < 0.05). In addition, the total analgesic consumption within 7 days in the MMH + ND-HAL group was less than in the MMH group (*P* < 0.05), as shown in Table 2. The incidence of postoperative bleeding, perianal incision edema, and acute urinary retention in the MMH + ND-HAL group was lower than that in the MMH group (*P* < 0.05), and neither group experienced anal stenosis or anal incontinence, as shown in Table 3.

**Table 2:** Postoperative visual analog scale for pain, total analgesic consumption over 7 days

Group	MMH + ND-HAL (*n* = 53)	MMH (*n* = 62)	t value	*P*-value
VAS (during first defecation)	4.15 ± 0.88	4.72 ± 0.96	−3.314	0.001
VAS (12 h)	3.35 ± 0.85	3.91 ± 0.83	−3.546	0.001
VAS (1 day)	3.39 ± 0.81	3.95 ± 1.07	−3.072	0.003
VAS (2 days)	3.13 ± 0.70	3.53 ± 0.91	−2.583	0.011
VAS (3 days)	2.81 ± 0.89	3.30 ± 0.75	−3.200	0.002
VAS (7 days)	2.00 ± 0.80	2.33 ± 0.76	−2.302	0.023
Total analgesic consumption within 7 days (g)	0.71 ± 0.20	0.80 ± 0.24	−2.111	0.037

Postoperative visual analog scale (VAS) for pain and total analgesic consumption over 7 days are presented as the mean ± standard deviation

*MMH + ND-HAL *Milligan-Morgan hemorrhoidectomy combined with non-Doppler hemorrhoidal artery ligation and perianal sealing, *MMH *Milligan-Morgan hemorrhoidectomy

**Table 3:** Postoperative complications

Group	MMH + ND-HAL (*n* = 53)	MMH (*n* = 62)	χ 2 value	*P*-value
Minor bleeding	1 (1.87%)	8 (12.90%)	-	0.037
Perianal incision edema	4 (7.55%)	14 (22.58%)	4.891	0.027
Acute urinary retention	2 (3.77%)	10 (16.13%)	4.667	0.031
Anal stenosis	0	0	-	-
Anal incontinence	0	0	-	-

Minor bleeding, perianal incision edema, acute urinary retention, anal stenosis, and anal incontinence are presented as N (percentage)

*MMH + ND-HAL *Milligan-Morgan hemorrhoidectomy combined with non-Doppler hemorrhoidal artery ligation and perianal sealing, *MMH *Milligan-Morgan hemorrhoidectomy

### Postsurgical recurrence and patient satisfaction at 12 months

To track the long-term outcomes and satisfaction of patients, follow-up visits were conducted by telephone or outpatient visits at 12 months after surgery. No patients in the MMH + ND-HAL group had recurrence (prolapse or bleeding), whereas 6 patients (9.68%) in the MMH group had recurrence (prolapse or bleeding) (*P* < 0.05). The satisfaction of the MMH + ND-HAL group (96.23%) was higher than that of the MMH group (82.54%) (*P* < 0.05), as shown in Table 4.

**Table 4:** Follow-up at 12 months

Group	MMH + ND-HAL (*n* = 53)	MMH (*n* = 62)	χ 2 value	*P*-value
Recurrence (prolapse or bleeding)	0 (0%)	6 (9.68%)	-	0.030
Patient satisfaction	51 (96.23%)	52 (82.54%)	4.667	0.031

Recurrence and patient satisfaction are presented as N (percentage)

*MMH + ND-HAL* Milligan-Morgan hemorrhoidectomy combined with non-Doppler hemorrhoidal artery ligation and perianal sealing, *MMH *Milligan-Morgan hemorrhoidectomy

## Discussion

At present, many treatment methods are available for hemorrhoids, including conservative treatment, instrument treatment, and surgical treatment, such as hemorrhoidectomy, stapler hemorrhoidectomy (SH), and Doppler-guided/-assisted HAL [14–16]. Although MMH has obvious postoperative pain, secondary bleeding, long recovery period, and other shortcomings, MMH is still the preferred surgical method for patients with grade III/IV hemorrhoids because of its exact curative effect, low recurrence rate, and cost-effectiveness [17–19].

Complications such as pain and bleeding after MMH, however, cannot be ignored. Haksal et al. [20] reported that among 206 patients who underwent MMH, 24 patients (12.9%) had bleeding symptoms within 7 days of the operation, and 2 patients underwent reoperations for bleeding. Even if multimodal pain management is implemented, poor postoperative pain relief is still a major problem. Gerbershagen et al. [21] performed a retrospective analysis of 115,775 patients from 578 surgical wards of 105 German hospitals and found that post-hemorrhoidectomy pain ranked 23rd out of 529 definitive surgical procedures. Gallardo et al. [22] found that 22.2% of patients after MMH had to take opioid analgesics. Therefore, to address these problems, we jointly adopted a new combined procedure method of MMH + ND-HAL to address some of the limitations of MMH and to meet the current requirements of minimally invasive surgery and rapid rehabilitation.

HAL blocks the blood supply of hemorrhoids by ligating the arteries and vessels supplying hemorrhoids, thus promoting hemorrhoid tissue atrophy and reducing hemorrhoid prolapse symptoms. Compared with hemorrhoidectomy, HAL has the advantages of less pain, less bleeding, and rapid recovery of working ability, but the recurrence rate is high [23, 24]. In this study, we found that the combination of HAL and MMH could take advantage of their respective advantages, improve efficacy, and reduce the recurrence rate. In HAL, a Doppler probe is used to locate and ligate the hemorrhoid artery, or the artery can be palpated and ligated with fingers without the help of the Doppler probe. Schuurman et al. [25] conducted a blinded randomized clinical trial of HAL with or without a Doppler transducer in patients with grade II and III hemorrhoids, and the results showed that HAL significantly reduced signs and symptoms of hemorrhoid disease, but the Doppler transducer did not contribute to this beneficial effect. Naqvi et al. [26] also reported that in terms of postoperative pain, bleeding, and patient satisfaction, HAL without Doppler guidance was an effective method to treat hemorrhoids. Therefore, compared with Doppler-guided HAL under direct vision, no significant difference has been observed in symptom improvement, pain, bleeding, prolapse, and other complications. Additionally, the equipment requirements are low and the operation is relatively simple. During the operation, because the purpose is to prevent rectal stenosis, it is important to be cautious of the HAL points, which should not be kept in the same plane, and the ligation points should not be too numerous (generally 3, 7, or 11 o’clock points).

Our study results show that compared with MMH, MMH + ND-HAL reduced intraoperative bleeding (*P* < 0.05), showing that ligation of hemorrhoidal arteries by ND-HAL can block the blood supply of hemorrhoids, thus reducing intraoperative bleeding. In terms of the VAS score at the first defecation, as well as that 12 h, 1 day, 2 days, 3 days, and 7 days after the operation, the MMH + ND-HAL group had lower scores than the MMH group (*P* < 0.05). The total analgesic consumption within 7 days in the MMH + ND-HAL group was lower than in the MMH group (*P* < 0.05), which indicated that MMH + ND-HAL effectively relieved the pain of surgical incision and reduced the consumption of painkillers. Postoperative bleeding, edema, urinary retention, anal stenosis and other complications are often associated with MMH. Our research results showed that both groups of patients did not have anal stenosis or anal incontinence. The incidence of postoperative bleeding, perianal incision edema, and acute urinary retention was lower in the MMH + ND-HAL group than in the MMH group (*P* < 0.05), which showed that the combined operation could reduce the incidence of postoperative bleeding and perianal incision edema, relieved the postoperative pain, made the urine excretion smooth, and reserved enough skin or mucosal bridges during the operation, which had little impact on anal function. Li et al. [27] also showed that compared with the traditional MMH, the MMH combined with HAL significantly reduced the amount of intraoperative bleeding and the incidence of postoperative bleeding and anal edema. After 12 months of follow-up, the recurrence rate of the MMH + ND-HAL group was lower than that of the MMH group (*P* < 0.05), and satisfaction was higher in the MMH + ND-HAL group than in the MMH group (*P* < 0.05). These results indicated that the combined operation on the basis of hemorrhoid resection and HAL could block the hemorrhoid blood supply [28–30], locally cause a chronic inflammatory reaction, produce tissue fibrosis, make the mucous membrane and submucosal supporting tissue adhesion and fixation, and reduce the postoperative recurrence rate, and improve patient satisfaction. At the same time, this combined operation can reduce postoperative pain, which may be related to lifting the rectal mucosa above the internal hemorrhoids after HAL, reducing the degree of prolapse of internal hemorrhoids, and thus reducing the surgical incision for MMH.

The limitations of this study include the small sample size, single-center study, short postoperative follow-up, and limited results. It is feasible to further expand the sample size, incorporate a multicenter study, and extend the follow-up time to improve findings.

## Conclusion

MMH + ND-HAL had fewer complications, lower recurrence rate, and higher patient satisfaction than MMH alone. Therefore, MMH + ND-HAL appears to be a satisfactory surgical procedure in the treatment of grade III/IV hemorrhoids.

## Data Availability

The datasets used during the current study are available from the corresponding author on reasonable request.
